# Molecular Insights on the Possible Role of Annexin A2 in COVID-19 Pathogenesis and Post-Infection Complications

**DOI:** 10.3390/ijms222011028

**Published:** 2021-10-13

**Authors:** Prakash Patil, Praveenkumar Shetty, Nithin Kuriakose, Pavan Gollapalli, Sukanya Shetty, Roopa Bhandary, Jamboor K. Vishwanatha, Sudeep D. Ghate

**Affiliations:** 1 Central Research Laboratory, KS Hegde Medical Academy (KSHEMA), NITTE (Deemed to be University), Mangalore 575018, India; prakashpatil@nitte.edu.in (P.P.); gollapallipavan@nitte.edu.in (P.G.); sudeep.ghate@nitte.edu.in (S.D.G.); 2 Department of Biochemistry, KS Hegde Medical Academy (KSHEMA), NITTE (Deemed to be University), Mangalore 575018, India; shettysukan@nitte.edu.in (S.S.); bhandarybio@nitte.edu.in (R.B.); 3 Division of Proteomics and Cancer Biology, Nitte University Center for Science Education and Research (NUCSER), NITTE (Deemed to be University), Mangalore 575018, India; nithinkpoulose@gmail.com; 4 Microbiology, Immunology & Genetics, Graduate School of Biomedical Sciences, University of North Texas Health Science Center, Fort Worth, TX 76107, USA; Jamboor.Vishwanatha@unthsc.edu

**Keywords:** Annexin A2, COVID-19, cytokine storm, infection, insulin resistance, pathogenesis, SARS-CoV-2, thrombosis

## Abstract

Severe acute respiratory syndrome-coronavirus 2 (SARS-CoV-2) has infected >235 million people and killed over 4.8 million individuals worldwide. Although vaccines have been developed for prophylactic management, there are no clinically proven antivirals to treat the viral infection. Continuous efforts are being made all over the world to develop effective drugs but these are being delayed by periodic outbreak of mutated SARS-CoV-2 and a lack of knowledge of molecular mechanisms underlying viral pathogenesis and post-infection complications. In this regard, the involvement of Annexin A2 (AnxA2), a lipid-raft related phospholipid-binding protein, in SARS-CoV-2 attachment, internalization, and replication has been discussed. In addition to the evidence from published literature, we have performed in silico docking of viral spike glycoprotein and RNA-dependent RNA polymerase with human AnxA2 to find the molecular interactions. Overall, this review provides the molecular insights into a potential role of AnxA2 in the SARS-CoV-2 pathogenesis and post-infection complications, especially thrombosis, cytokine storm, and insulin resistance.

## 1. Introduction

Severe acute respiratory syndrome-coronavirus 2 (SARS-CoV-2), a single known causative agent of coronavirus disease 2019 (COVID-19), has infected more than 235 million people worldwide. It has caused severe social and economic disruption with mortality rate of around 2.2%, accounting for the deaths of over 4.8 million individuals [1,2,3]. The highly recombinogenic SARS-CoV-2 is the seventh known coronavirus to infect humans since the first human coronaviruses that were described in 1960s [4,5,6]. There are currently no clinically proven antivirals; however, a few vaccines with limited clinical efficacy have been developed for the treatment of COVID-19. In addition, various repurposed drugs, such as nucleoside analogues like remdesivir, translation inhibitors, compounds like hydroxychloroquine, dexamethasone, and dietary supplements like vitamins, minerals, folate, butyrate, etc. are being used in the treatment of COVID-19 [7,8]. Ongoing efforts are being made to develop drugs and vaccines all over the world but are being hampered by the frequent outbreaks of mutant SARS-CoV-2 [9]. Moreover, there is a lack of knowledge about the molecular mechanisms involved in SARS-CoV-2 replication as well as its interaction with human host cells, except angiotensin-converting enzyme 2 (ACE2), a cell membrane receptor protein for the viral entry [10,11,12]. ACE2, a component of the renin-angiotensin-aldosterone system, catalyzes the conversion of angiotensin II to angiotensin-(1–7) and angiotensin I to angiotensin-(1–9), lowering blood pressure [13,14]. ACE2 receptor expression is found in a wide range of cells, including vascular endothelial cells, smooth muscle cells, nasal and oral mucosa, enterocytes, type II alveolar pneumocytes, and others, indicating the tissue susceptibility to SARS-CoV-2 infection [15,16]. It has recently been discovered that high levels of circulating ACE2 in severe COVID-19 patients are linked to a higher risk of serious cardiovascular events such heart failure and aortic stenosis [17].

SARS-CoV-2 is an enveloped betacoronavirus with a positive-sense, single-stranded RNA genome belonging to the family coronaviridae [18,19]. SARS-CoV-2 has a genome size of 29.9 kb, sharing 79.5% and 96.2% identity with SARS-CoV and bat CoV RaTG13, respectively [20]. The viral genome encodes for 29 proteins that include 16 non-structural proteins including an error-prone RNA-dependent RNA polymerase (RdRp) for viral replication, nine accessory proteins for countering host immune responses, and four structural proteins comprising the nucleocapsid (N) protein that encapsulates the RNA genome and three membrane proteins: the spike (S)-glycoprotein, the matrix (M) protein, and the envelope (E) protein [6,21,22,23]. Approximately 40% of these SARS-CoV-2 proteins were engaged in vesicle trafficking pathways connected with endomembrane compartments for coronavirus replication [24,25,26]. All coronavirus mRNAs rely on cap-dependent translation to produce their proteins [27,28]. However, the basic functional features of these viral proteins, as well as the role of their interacting host proteins in virus multiplication and survival, are yet unknown.

The S-glycoprotein is a highly glycosylated homotrimer made up of S_1_ and S_2_ subdomains. The S_1_ subdomain encodes the receptor binding domain and is responsible for binding to host cell receptor, whereas the S_2_ subdomain mediates fusion of the viral and host cell membranes by forming a six-helical bundle via the two-heptad repeat domain [13,29,30]. A transmembrane metallopeptidase, ACE2, was initially identified as a SARS-CoV-2 receptor; however, subsequent researchers found that neuropilin-1 is also a host component that aids infectivity [10,12,31,32,33,34]. However, recent studies show that SARS-CoV-2 enters cells via clathrin-mediated endocytosis, implying that this pathway is an important part of virus infectivity [35]. Furthermore, it has been observed that S-glycoprotein has a furin cleavage site at the S_1_/S_2_ boundary and mediates the cleavage through the type II transmembrane serine protease TMPRSS2 at S_2_ site to activate the membrane fusion [10]. This fusion is a molecular mimic of SNARE-mediated cellular membrane fusion, and it generates a channel that allows the virus’s RNA and RNA-associated nucleocapsid proteins to gain access to the cytosol, causing infection [36]. As a result, the viral and host cell membranes’ lipid bilayer composition, particularly the lipid rafts, is crucial for virus membrane fusion and entry into the host cell [37]. Therefore, the host cell membrane’s lipid rafts, which house multiple transmembrane proteins involved in receptor signaling and trafficking via the secretory and endocytic pathways, could serve as hotspots for virus entry [35,37,38].

A pleiotropic protein, Annexin A2 (AnxA2), is a calcium-dependent protein implicated in a variety of membrane-related biological activities including cell-cell adhesion, exocytosis, endocytosis, and the creation and stabilization of lipid rafts [38,39]. Moreover, AnxA2 has been reported to be involved in attachment, internalization, replication, and pathogenesis of several viruses including SARS-CoV [40,41]. Recent investigations identified the presence of anti-AnxA2 autoantibodies in hospitalized COVID-19 patients [42]. Thus, in this review, we specifically considered the evidence from the published literature and in silico docking analysis data to provide the molecular insights into the potential role of AnxA2 in the SARS-CoV-2 pathogenesis and post-infection complications, especially thrombosis, cytokine storm, and hyperglycemia.

## 2. AnxA2 Role in SARS-CoV-2 Pathogenesis

**Figure 1:**
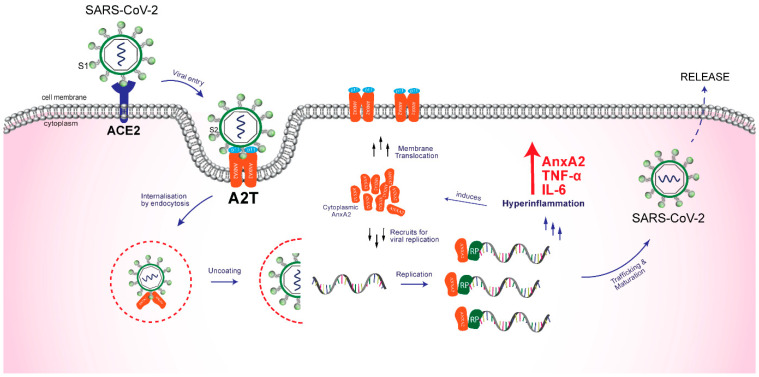
A schematic illustration of the possible functional role of human AnxA2 in internalization and replication of SARS-CoV-2. The process starts by the recognition of host cell ACE2 receptor by viral spike glycoprotein S1, which is followed by the spike glycoprotein S2 interaction with A2T at the membrane surface. After the furin cleavage at S1/S2 boundary, the virus gets endocytosed via clathrin/SNARE-mediated pathway. Furthermore, AnxA2 interacts with viral non-structural proteins like RNA-dependent RNA polymerase for replication and helps in trafficking, maturation, and viral release. AnxA2, being a proinflammatory upstream regulator, results in inflammatory surge and cytokine storm. ACE2, Angiotensin converting enzyme 2; AnxA2, Annexin A2; A2T, Annexin A2 Tetramer; IL-6, Interleukin 6; p11, Protein S100A10; RP, Replication protein; S1, Spike glycoprotein 1; S2, Spike glycoprotein 2, and TNF-α, Tumor necrosis factor alpha.

AnxA2 is a phospholipid-binding 36-kDa protein that exists as a monomer in the cytoplasm or as a heterotetramer in the extracellular plasma membrane, generated by the non-covalent interaction of two AnxA2 molecules with two S100A10 (p11) molecules [43,44]. The AnxA2 tetramer (A2T) acts as a receptor for tissue plasminogen activator (tPA), which converts plasminogen into plasmin, which is involved in fibrinolysis [45]. In addition to its RNA binding protein activity, AnxA2 operates as an upstream signaling molecule for multiple downstream cell signaling pathways that are involved in the control of various cellular processes such as cell proliferation, metastasis, angiogenesis, and inflammation [38,46,47]. With this background, we have tried to gather evidence to support the possible role of AnxA2 in SARS-CoV-2 pathogenesis including attachment, internalization, trafficking, and replication (Figure 1).

### 2.1. Role in Attachment and Internalization of the Virus

Cellular attachment and penetration into the targeted host, which is dependent on host cell components and processes such as cellular receptors, is a critical part of any viral life cycle. AnxA2 has been connected to a number of membrane-related host-virus interactions, including virus attachment, internalization, and trafficking (Table 1). The pseudorabies virus (PRV) infection induces the AnxA2 production in axons and exploit it for the efficient retrograde transport in the peripheral nervous system neurons. [48]. A plasma membrane-associated receptor AnxA2 also interacts with enterovirus type 71 (EV71) VP1 to promote virus attachment and infection [49,50,51]. AnxA2 binds to viral membrane anionic phospholipids of cytomegalovirus (CMV) on the cell surface of endothelial cells in a calcium-dependent manner to fuse with the host cell membrane, which varies on the cell type [52,53,54]. The L2 protein of the human papillomavirus (HPV) interacts with the p11 component of A2T to aid in HPV internalization, indicating that both the tetramer and monomeric forms of AnxA2 play a vital role in HPV infection by preventing the lysosomal breakdown [55,56]. Furthermore, the human immunodeficiency virus (HIV) envelope protein’s phosphatidylserine binds to AnxA2 on the cell membrane, which serves as a cellular cofactor supporting the macrophage HIV-1 infection [57,58]. Early infection with dengue virus serotype 2 (DENV2) produces an increase in the synthesis of AnxA2, a protein involved in cytoskeletal rearrangements that could also serve as a host cell factor for viral internalization [59]. The respiratory syncytial virus (RSV) glycoproteins G and F are involved in cell attachment and fusion. RSV G protein interacts with L-selectin and AnxA2, which are expressed on the surfaces of leukocytes and epithelial cells, respectively, and RSV infection promotes AnxA2 expression on the epithelial cell surface [60].

**Figure 2:**
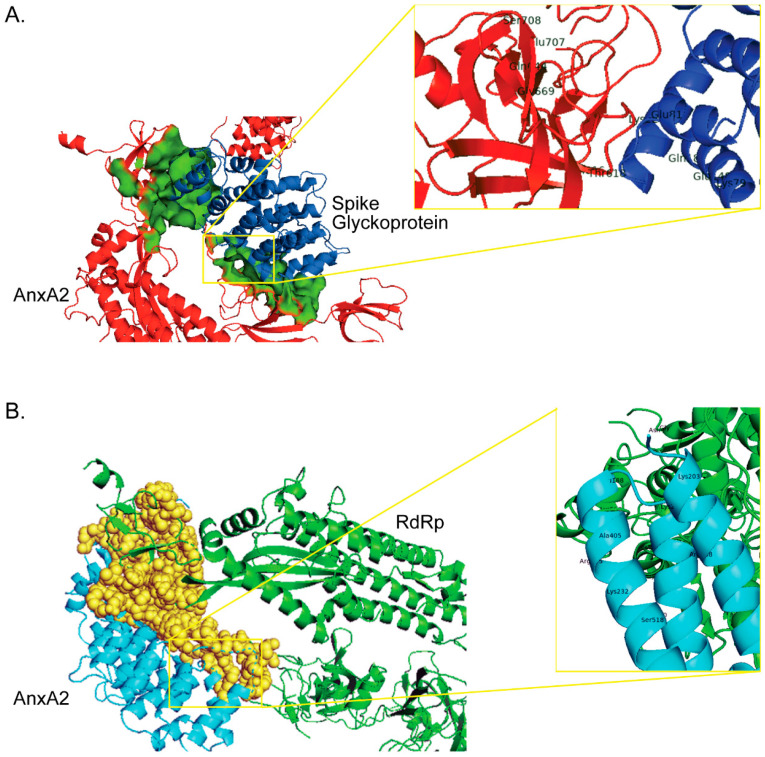
The low-energy binding conformations generated by molecular docking of human AnxA2 with spike glycoprotein (**A**) and RNA-dependent RNA polymerase (**B**) of SARS-CoV-2. The close-up image shows the detailed view of the interacting residues in the region where human AnxA2 bound to spike glycoprotein and RdRp of SARS-CoV-2.

To understand the molecular interaction between human AnxA2 (PDB entry: 6M71; resolution: 2.90 Å) with the SARS-CoV-2 spike glycoprotein (PDB entry: 6LZG; resolution: 2.50 Å), molecular docking studies were performed using the fully automated web-based platform, ClusPro v2.0 (http://cluspro.bu.edu/ (accessed on 13 September 2021)) that executes rigid body docking, when a partner protein in a complex is structurally flexible [61,62,63]. This returned with the top models based on energy and cluster size that are having interactions within the same sphere of binding site for the docking complexes. Further, the docked conformations were filtered based on surface complementarities, which were ranked according to their clustering parameters so as to sort those with highest free energy values, ΔG (kcal/mol) as obtained by smoothening their local minima [63,64]. Among the top generated docking conformations having interactions in the same region of proteins, a model interaction with the lowest docking energy of −889.3 and largest cluster size of 24 members was further analyzed using PyMol v2.4.0 program [65]. It was observed that there were 12 hydrogen bonds and 2 salt bridges between the interacting amino acid residues in the C-terminal region of SARS-CoV-2 spike glycoprotein (Arg1107, Thr1077, Ser708, Asn616, Thr618, Glu707, Gln644, and Gly669) and human AnxA2 (Glu188, Tyr187, Lys226, Gln75, Lys79, Gln187, Glu81, and Glu148) (Figure 2A and Figure S1A). The hydrogen bond interactions determine the structural integrity of many biological molecules, and are crucial for protein folding and binding. According to Janin et al. [61], an interface with at least ten hydrogen bonds has enough enthalpy to establish binding affinities of up to 10^−14^ M, indicating that the human AnxA2 can form a stable complex of protein-protein interaction with viral spike glycoprotein.

### 2.2. Role in Replication and Release of Virus

Viruses must take over and reprogram host cells in order to successfully create their progeny in the infected host. This coordinated complex process relies on the host cell components to facilitate the viral uncoating, integrating into host genome, and protein expression to replicate and release the viral particle. During the process, AnxA2 is utilized by measles virus (MV), hepatitis C virus, (HCV), influenza A virus (IAV), hepatitis B virus (HBV), and human immunodeficiency virus (HIV) during the assembly, maturation, and replication (Table 2). In HeLa cells, the MV matrix protein helps attach the viral capsid to the viral envelope so that it fuses with the host cell membrane, and it traffics through monomeric AnxA2 rather than A2T [66]. The lipid rafts have been shown to have a role in the development of crude replication complexes, in which HCV replication is mediated by the non-structural protein NS5A, which recruits AnxA2 [67,68]. However, AnxA2’s direct contact with HCV’s NS5B lowers NS5B’s polymerase activity and RNA binding ability [69]. AnxA2 and A2T have been observed on the IAV H1N1 viral envelopes, with A2T being a plasminogen receptor involved in the conversion of plasminogen to plasmin, which is required for IAV replication [70]. Furthermore, H5N1’s non-structural protein NS1 interacts with AnxA2, promoting viral reproduction in host cells. However, silencing AnxA2 causes a decrease in the expression of viral proteins Hemagglutinin (HA) and Matrix (M), which are required for viral attachment and infection [71]. H5N1 infection, on the other hand, reduces AnxA2 expression, and lowered AnxA2 expression increases H5N1 spread [72]. AnxA2 in lipid rafts containing ganglioside M1 interacts with viral p41Gag protein and PIP2 on the plasma membrane, as well as with viral polyprotein precursor protein p55Gag on the late endosomal membrane, where HIV assembly and replication occur [73,74]. This is disputed because it is only seen in macrophages produced from monocytes [58]. HBV polymerase interacts with p11, a subunit of the A2T, which mediates HBV polymerase recruitment to nuclear bodies of promyelocytic leukemia protein in a Ca^2+^-dependent way [75].

In our protein–protein docking studies performed as explained above between the human AnxA2 (PDB entry: 6M71; resolution: 2.90 Å) and SARS-CoV-2 RdRp (PDB entry: 2HYW; resolution: 2.10 Å), the top model conformation with lowest docking energy of −738.1 and highest cluster size of 11 members was identified. The conformation has 10 hydrogen bonds between the interacting amino acid residues in the C-terminal region of SARS-CoV-2 RdRp (Gly337, Asn404, Val405, Ser518, Lys508, Arg365, Asp336, Tyr374, and Lue401) and human AnxA2 (Lys203, Arg308, Thr187, Lys226, Glu188, Arg195, Arg230, and Lys232) (Figure 2B and Figure S2B). These data show that the human AnxA2 can also form a more stable interaction protein complex with viral RNA-dependent RNA polymerase, indicating their role in viral replication, maturation, and release into the host cell.

## 3. AnxA2 Role in Cytokine Storm

The recent literature reviews on several studies that looked at the cytokine profiles of COVID-19 patients indicate that the ‘cytokine storm’ was connected to lung injury, multi-organ failure, and a dismal prognosis [67,68,69,70]. This cytokine storm phenomenon represents the release of a large number of pro-inflammatory cytokines as a hyperactive immunological response triggered by SARS-CoV-2 infection [76,77,78,79]. Among the pro-inflammatory molecules, Fang et al. identified an upregulated expression of Ca^2+^-dependent binding protein AnxA2 by SARS-CoV associated cytokines and the cross-reactivity of anti-SARS-CoV Spike domain-2 antibodies to AnxA2 [41]. AnxA2, being a pro-inflammatory receptor protein, its enhanced synthesis could trigger hyper-immunogenicity, resulting in autoantibodies against it. Additionally, it has been observed in the hospitalized COVID-19 patients that there is a massive production of pro-inflammatory cytokines including IL-6, IL-1β, and TNFα, whose production is known to be stimulated by the concentrated AnxA2 at the plasma membrane in the heterotetramer form as A2T [80,81,82]. The intracellular AnxA2 binds to p50 subunit of pro-inflammatory transcription factor NF-κB, leading to increased transcriptional activity and the expression of adrenomedullin, CSF2 (GM-CSF), F3, IL-1B, IL1R2, IL-6, CD40, and GADD45B genes [83]. Activated coagulation factor X induces AnxA2 dependent cytokine production in airway smooth muscle cells and may cause fibrosis in lungs [84,85]. AnxA2 also regulates the production of inflammatory cytokines (IL-6 and IL-8) by airway smooth muscle cells in response to plasminogen stimulation by activating the ERK1/2, p38MAPK, and PI3K/Akt downstream pathways [86].

The local production of AnxA2 within the tissues promotes the complement-mediated bacterial clearance by suppressing the factor H, a circulating protein that regulates the alternative pathway of complement. [87]. In colonic epithelial cells and monocytes, AnxA2 interacts directly with ADAM17 and mediates the ectodomain shedding of TNF-α [88]. A2T activates and stimulates the production of inflammatory cytokines via a TLR 4-dependent mechanism [89]. The increased production of inflammatory cytokine IL-1β by the gram-negative bacteria *Vibrio vulnificus* promoted the phagocytic death via NF-κB regulation mediated by AnxA2 [90,91]. Increased expression and raft distribution of AnxA2 are stimulated by SARS-CoV-induced cytokines IL-6 and IFN-γ, as well as enhanced anti-S2 antibody binding activity, resulting in thrombotic complications and lung inflammation, leading to a severe form of SARS-CoV infection [41]. Moreover, recent reports have also shown that the elevated levels of anti-AnxA2 antibodies are observed among COVID-19 patients causing cytokine storm that may finally lead to systemic thrombosis, cell death, and non-cardiogenic pulmonary oedema [42,92,93]. This could be due to the hyper-immunogenicity of AnxA2, as it has been found to be an autoantigen in patients with anti-phospholipid syndrome and lung cancer [94,95]. In addition, according to our findings, AnxA2 acts an upstream regulator of pro-inflammatory cytokines, such as IL-6, IL-1β, and TNFα, and also play a reciprocal regulation with anti-inflammatory AnxA1 in the pathogenesis of rheumatoid arthritis [96]. Furthermore, a glucocorticoid dexamethasone treatment, in severe COVID-19 patients, is known to induce anti-inflammatory AnxA1 and also enhances its translocation to cell membrane, could reduce the membrane deposition of AnxA2, making it unavailable for the virus entry and replication [97].

## 4. AnxA2 Role in Thrombosis

According to recent clinical data, COVID-19 is linked to a high risk of thrombotic consequences such as microvascular thrombosis, venous thromboembolic illness, and stroke [98,99,100,101,102]. These thrombotic events occur in up to one-third of COVID-19 patients and are linked to more severe illness and mortality [103]. Recent evidence suggests that decreased plasma fibrinolysis is a risk factor for venous thromboembolism, and that cell surface tPA receptor, especially AnxA2 is involved in this hypofibrinolysis [104,105]. However, previous research has linked the release of anti-phospholipid antibodies, which is seen in anti-phospholipid syndrome (APS), to arterial and venous thrombosis as a possible risk factor. The majority of these antibodies recognize phospholipid-binding proteins such as β2 Glycoprotein I (β2GPI) and prothrombin, which activate endothelial cells when β2GPI binds with AnxA2 with high affinity. The profibrinolytic tPA-plasminogen receptor, AnxA2 complex on the cell surface facilitates plasmin production and triggers endothelial cell activation [94,106,107,108].

In fact, the activation of vascular endothelial cells in the inner lining of blood vessels lead to the loss of anti-coagulant and anti-thrombotic properties that are vital for blood fluidity [109]. The AnxA2 forms a multiprotein signaling complex with toll-like receptor 4, calreticulin, and nucleolin, which mediate anti-β2GPI antibodies induced endothelial cell activation, leading to thrombosis associated with APS [110,111]. Intravenous injection of recombinant AnxA2 dramatically reduces thrombus development in a rat model of embolic stroke without changing hemostatic parameters [112]. Additionally, the role of AnxA2 in maintaining the endothelial cell junctions around lung microvasculature and preventing pulmonary edema, especially in response to hypoxia, has recently been identified [113]. Given its critical protective roles, the levels of anti-AnxA2 antibodies among hospitalized COVID-19 patients highly predicted the mortality, indicating the importance of AnxA2 in the pathophysiology of SARS-CoV-2 infection [42]. In severe COVID-19 patients, an apparent hypofibrinolysis has been observed, which could be related to the ceaseless engagement of AnxA2 with viral replication, rendering it unavailable for its typical function of tPA mediated conversion of plasminogen to plasmin, a major participant in fibrinolysis [104,114]. As hypofibrinolytic and hyperthrombin production have been linked to SARS-CoV-2-related thrombosis, the tPAs or AnxA2 agonists that can induce thrombolysis may be beneficial for treating pulmonary embolism (PE) and hemodynamic instability [115]. Therefore, the use of tPA in the treatment of critically-ill COVID-19 patients with PE and acute respiratory distress syndrome (ARDS) may be advantageous [116,117,118]. Furthermore, the broad spectrum immunosuppressant drug dexamethasone treatment given to critically ill COVID-19 patients may be associated with the risk of venous thromboembolism because corticosteroids are known to induce anti-inflammatory AnxA1, recruiting to the membrane surface results in lowered translocation of AnxA2 to the cell membrane, which could precipitate into thrombosis [119].

## 5. AnxA2 Role in Insulin Resistance

According to recent evidence, diabetes mellitus and hyperglycemia are the predictors of poor prognosis and increased risk of death among COVID-19 patients [120,121,122,123,124]. Moreover, a new-onset of acute hyperglycemia has been observed in severe COVID-19 patients, which can exacerbate the existing diabetes, resulting in increased insulin resistance, hyper-inflammation, and a lower probability of survival [125]. In the literature, there are several reviews on the proposed concept that explains the sudden onset of hyperglycemia in individuals with severe COVID-19 infection. The most widely debated theory is that SARS-CoV-2 infects the pancreas via ACE2 interaction on pancreatic β cells, which then impairs endogenous insulin synthesis by altering insulin receptor signaling, resulting in acute hyperglycemia and devastating hyperinflammation [126,127,128].

Hyperglycemia and diabetes mellitus reduce fibrinolytic activity on the endothelial cell surface by lowering tPA and plasminogen expression and increasing plasminogen activator inhibitor-1 expression. Hyperglycemia also causes hyper-coagulation by increasing the levels of advanced glycation end-products modified forms of cellular and membrane AnxA2 [129,130]. In a high-fat diet induced IR mouse model study, silencing AnxA2 attenuated the obesity-induced insulin resistance and inflammation through the process of p50 nuclear translocation of the NF-κB signaling pathway [131]. Furthermore, AnxA2 interacts with galectin-3 at the cell surface, which directly induces cellular insulin resistance and associated inflammation under obese conditions by impairing insulin signaling [132,133,134]. As evidenced by recent studies, the anti-AnxA2 antibodies detected in severely infected COVID-19 patients suggest that AnxA2 expression is increased following SARS-CoV-2 infection, which could disrupt insulin signaling via the galectin-3 or NF-κB pathways, resulting in insulin resistance and hyperglycemia [42,135,136]. Furthermore, the viral infection-mediated increased production of AnxA2 interacts with galectin-3, which can bind directly to the insulin receptor and prevent it from being activated by insulin [132,137]. This suppresses the down-stream signaling of IRS1 tyrosine phosphorylation, PDK1 activation, and AKT phosphorylation, resulting in insulin-dependent glucose uptake and translocation of GLUT4 inhibition, and ultimately results in hyperglycemia [138].

## 6. Summary and Future Perspectives

**Figure 3:**
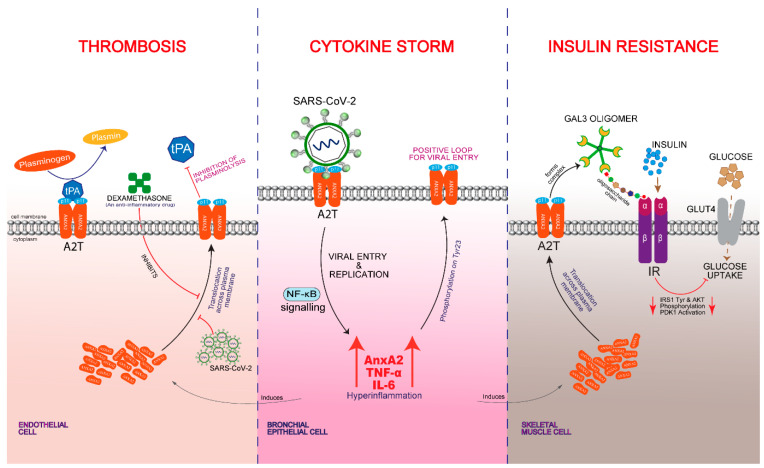
The proposed model for the pathophysiological involvement of human AnxA2 interaction with SARS-CoV-2 spike glycoprotein S2 at the membrane surface. Upon host cell infection, virus induces the production of AnxA2 and utilizes it for internalization and replication, triggering an aggressive immune response that results in the release of a large amount of pro-inflammatory cytokines including autocrine AnxA2, and IL-6, IL-1β, and TNF-α, resulting in a cytokine storm. In severe COVID-19 patients, an apparent hypofibrinolysis has been observed, which could be related to the ceaseless engagement of AnxA2 with viral replication, rendering it unavailable for its typical function of tPA mediated conversion of plasminogen to plasmin, a major participant in fibrinolysis. In addition, a methylprednisolone or dexamethasone, an anti-inflammatory or immunosuppressant drug given to hospitalized COVID-19 patients with pneumonia, induces anti-inflammatory AnxA1 and recruits it to the cell membrane, resulting in decreased membrane deposition of AnxA2 that could further impede the plasminolysis. Furthermore, the glycosylated AnxA2 at the membrane surface interacts with galectin-3 that can bind directly to the insulin receptor and blocks its activation and inhibits down-stream signaling such as IRS1 tyrosine phosphorylation, PDK1 activation, and AKT phosphorylation. Subsequently, it leads to the inhibition of insulin-dependent glucose uptake and translocation of GLUT4, resulting in hyperglycemia. ACE2, Angiotensin converting enzyme 2; AKT, Protein kinase B; AnxA2, Annexin A2; A2T, Annexin A2 Tetramer; GAL3, Galectin-3; GLUT4, Glucose transporter type 4; IL-6, Interleukin 6; IR, Insulin receptor; IRS1, Insulin receptor substrate 1; NF-κB, Nuclear factor kappa B; p11, Protein S100A10; PDK1, Pyruvate dehydrogenase kinase 1; TNFα, Tumor necrosis factor alpha; and tPA, Tissue plasminogen activator.

Multiple evidences in this review, including the detection of anti-AnxA2 antibodies in COVID-19 patients, suggests that AnxA2 has an implicating role in SARS-CoV-2 pathogenesis and post-infection complications (Figure 3). In addition to its wide range of functions including plasminolysis, we have established in our earlier studies that the AnxA2 has importance in EGFR downstream signaling and its interacting partner galectin-3 regulation [137,139]. Furthermore, we also elucidated that AnxA2 may function as an upstream pro-inflammatory regulator and have reciprocal regulation with AnxA1 in inflammatory conditions like rheumatoid arthritis [96]. According to our preliminary protein docking investigations, the AnxA2 might interact with S2 and RdRp proteins of SARS-CoV-2 to aid in internalization and replication. After attachment to the host cell, SARS-CoV-2 utilizes the membrane bound AnxA2 for internalization, decreasing the A2T mediated cell surface plasminolysis, and leading to the intravascular thrombosis in COVID-19 patients. The virus will then use the cytoplasmic monomeric AnxA2 for replication in the infected cell, resulting in a devastating inflammatory surge and cytokine storm. The viral mediated AnxA2 induction and its membrane translocation might recruit galectin-3 to bind insulin receptor, leading to insulin resistance.

The currently available treatment options for COVID-19 includes broad spectrum antivirals, nutritional immune boosters, immunosuppressants or corticosteroids, etc. [140,141,142]. Though there is insufficient data to support the beneficial effects of nutritional supplements such as vitamins, minerals, fatty acids, herbs, etc. are being recommended for proper immune function [143]. In addition, we recently made a detailed review and proposed a dietary supplement, sodium butyrate or butyric acid, which has an anti-inflammatory effects, could be an effective therapeutic strategy for COVID-19 prevention [144]. However, there is a general dearth of particular antiviral drugs that target the virus rather than simply providing symptomatic relief. The vaccine research, despite moving at a rapid pace and yielding more than a dozen vaccinations, has been unable to keep up with the virus’s global mutation rate [145,146]. Furthermore, the vaccines’ efficacy in offering protection against existing mutations or forthcoming variations is still unknown [147]. As a result, there is a great demand for novel drugs and strategies that can either prevent the virus entry or replication inside the host cells. Currently, drug repurposing and in silico docking studies are being pursued in order to provide therapeutic insights into mechanisms that could be effective in combating the virus [148]. Given the situation, AnxA2 could be explored as a potential therapeutic target to combat SARS-CoV-2 infection because it has been already tested as an antigenic target for T cell immunotherapy in pancreatic cancer and also known to serve as a target for developing anti-angiogenic drug to prevent cancer aggressiveness [149,150,151]. Furthermore, we believe that multidisciplinary research is needed to fully understand these mechanistic pathways and to confirm their therapeutic implications against this viral infection.

## Figures and Tables

**Table 1:** The viruses interacting with AnxA2 for cell attachment, entry and internalization.

Virus	Family & Genus	Genome			AnxA2 Function	Ref.
		Strand	Type	Size		
PRV Pseudorabies virus	Herpesviridae Varicellovirus	Double, Linear positive	DNA, envelope	143 Kb	Involved in retrograde trafficking of PRV in axons	[48]
CMV Cytomegalovirus	Herpesviridae Cytomegalovirus	Double, Circular	DNA, envelope	200 Kb	Bridge virus to host cell membrane and enhances infection	[52,53,54]
HPV Human Papillomavirus	Papillomaviridae Alphapapillomavirus	Double, Circular	DNA, non-envelope	7.9 Kb	Interact with viral L2 capsid protein for internalization and infection	[55,56]
EV71 Enterovirus type 71	Picornaviridae Enterovirus	Single, Linear positive	RNA, non-envelope	7.5 Kb	Interact with viral VP1 for host cell entry	[49,50,51]
DENV Dengue virus	Flaviviridae Flavivirus	Single, Linear positive	RNA, envelope	10.7 Kb	Requires active filopodia formation for a successful DENV infection	[59]
RSV Respiratory syncytial virus	Pneumoviridae Orthopneumovirus	Single, Linear negative	RNA, envelope	15 Kb	Potential epithelial cell receptor for RSV infection	[60]
HIV Human Immunodeficiency Virus	Retroviridae Lentivirus	Single, Linear positive	RNA, envelope	9.2 Kb	Regulates HIV1 infectivity in cell dependent manner	[57,58]
SARS-CoV Severe acute respiratory syndrome coronavirus	Coronaviridae Betacoronavirus	Single, Linear positive	RNA, envelope	30 Kb	Colocalised with S2 protein of SARS-CoV, role in internalization	[41]

**Table 2:** The viruses interacting with AnxA2 for their replication, trafficking and release out of cell.

Virus	Family & Genus	Genome			AnxA2 Function	Ref
		Strand	Type	Size		
MV Measles Virus	Paramyxoviridae Morbillivirus	Single, Linear negative	RNA, envelope	16Kb	Interacts with M protein of the virus to mediates the cellular localization	[66]
HCV Hepatitis C virus	Flaviviridae Hepacivirus	Single, Linear positive	RNA, envelope	9.6Kb	Interact with NS5A/B protein of virus involved in assembly and replication	[67,68,69]
IAV Influenza A viruses	Orthomyxoviridae Alphainfluenzavirus	Single, Linear negative	RNA, envelope	13.6Kb	Activation of plasminogen helps the virus to replicate	[70,71,72]
HBV Hepatitis B Virus	Hepadnaviridae Hepadnavirus	Double, Linear negative	DNA, envelope	3.2Kb	In modulating HBV Pol function for intracellular viral replication	[75]
HIV Human Immunodeficiency Virus	Retroviridae Lentivirus	Single, Linear positive	RNA, envelope	9.2Kb	Interact with HIV-1 Gag binding protein for replication in monocyte-derived macrophages	[73,74]

## Supplementary Materials

The following are available online at https://www.mdpi.com/article/10.3390/ijms222011028/s1.
