# Supplementary material for: Molecular Insights on the Possible Role of Annexin A2 in COVID-19 Pathogenesis and Post-Infection Complications
Source: Int J Mol Sci. 2021 Oct 13;22(20):11028. doi: 10.3390/ijms222011028 (PMC8538098; doi:10.3390/ijms222011028)
Supplement: Supplementary file 1 [file ijms-22-11028-s001.zip › ijms-1368739-supplementary.pdf]

Supplementary File

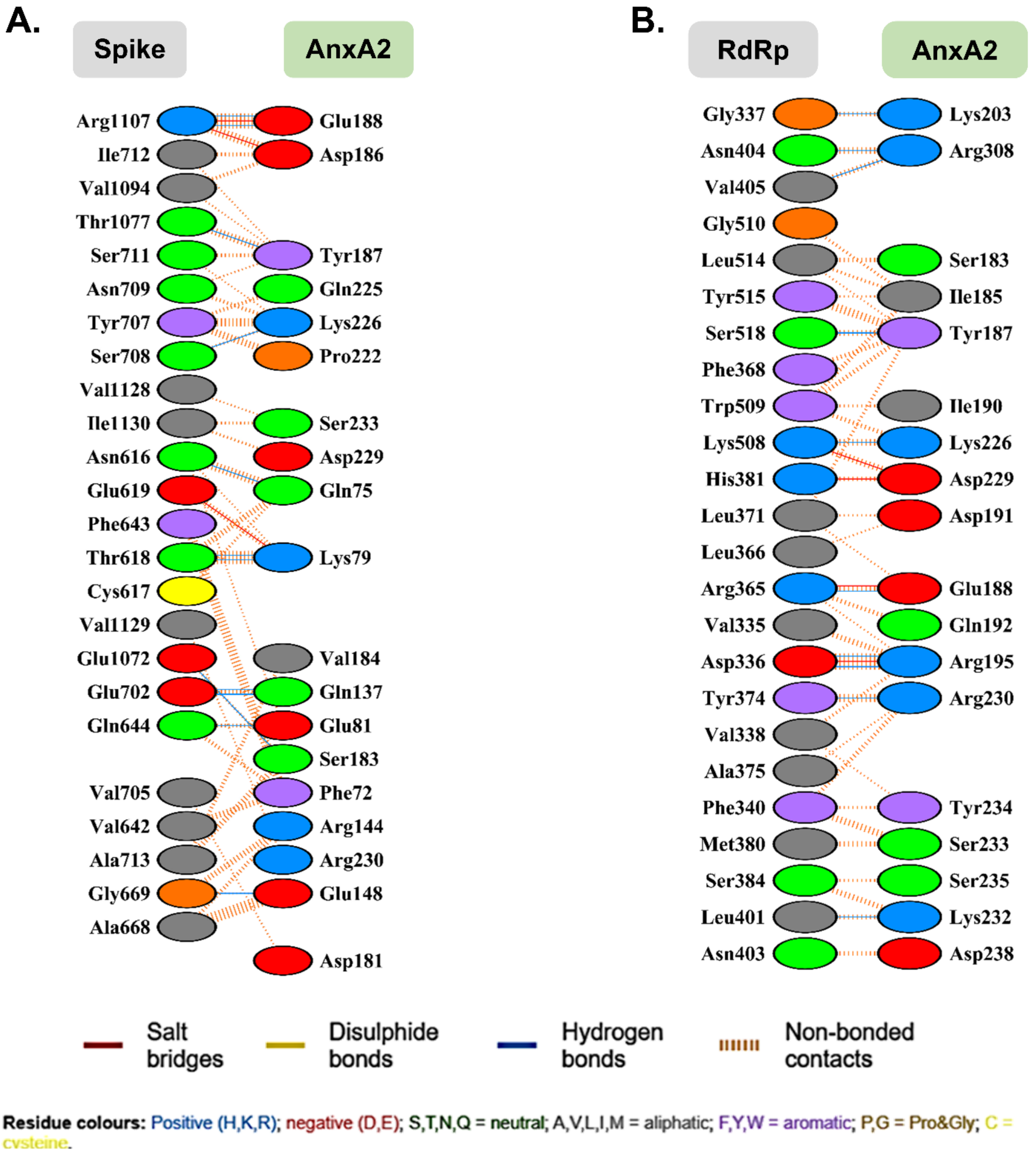

Figure S1. The interacting residues of human AnxA2 bound to spike glycoprotein and RdRp of SARS-CoV-2.
